# Hemiphlegia from Cerebral Hemorrhage in Cases Where There Is Valvular Disease of the Heart

**Published:** 1871-03

**Authors:** 


Hemiphlegia from Cerebral Hemorrhage in Cases where there is Valvular Disease of the Heart.—The mere fact that hemiphlegia has occurred in a person, even a young person, the subject of valvular disease of the heart, must not alone lead one to the diagnosis of embolism. The mode of onset is the important matter in the diagnosis of the nature of the lesion in all cases of hemiphlegia. If we learn that the patient had been deeply insensible for many hours, or, more generally, if he had been for some time in the "apoplectic condition," the diagnosis of cerebral hemorrhage is at least as likely as that of plugging, notwithstanding that there is valvular disease. The bleeding is from an aneurism of the middle cerebral artery, or from a branch of it. It is true that, as a rule, such aneurisms burst outside of the brain, and do not produce hemiphlegia, but now and then they imitate, so to speak, ordinary cerebral hemorrhage, and break up the motor track. Dr. Hughlins Jackson's observations confirm the opinion of Dr. John W. Ogle and Dr. Church as to the association of aneurisms of the larger cerebral arteries with vegetations on the heart's valves. Dr. Hughlins Jackson thinks that the kind of defect of speech which sometimes accompanies hemiplegia is useful evidence as to the *pathological* nature of the lesion. *Loss* of speech—loss excepting the patient's stock word or phrase—is in favor of hemorrhage. Still, plugging will produce complete and permanent loss of speech. If the patient recover, and if during the recovery he make frequent mistakes in words—the so-called loss of memory for words—articulating them clearly, Dr. Hughlins Jackson thinks the diagnosis of plugging more warrantable than that of clot.—*British Medical Journal*, Oct. 29, 1870.

